# Dehydration of d-fructose to 5-hydroxymethyl-2-furfural in DMSO using a hydrophilic sulfonated silica catalyst in a process promoted by microwave irradiation

**DOI:** 10.1038/s41598-020-80285-2

**Published:** 2021-01-21

**Authors:** Sandro L. Barbosa, Milton de S. Freitas, Wallans T. P. dos Santos, David Lee Nelson, Stanlei I. Klein, Giuliano Cesar Clososki, Franco J. Caires, Adriano C. M. Baroni, Alexandre P. Wentz

**Affiliations:** 1 Department of Pharmacy, Universidade Federal dos Vales do Jequitinhonha e Mucuri-UFVJM, Campus JK, Rodovia MGT 367 - Km 583, nº 5.000, Alto da Jacuba, Diamantina, MG CEP 39100-000 Brazil; 2 Department of General and Inorganic Chemistry, Institute of Chemistry, São Paulo State University-Unesp, R. Prof. Francisco Degni 55, Quitandinha, Araraquara, SP CEP-14800-900 Brazil; 3 Department of Physics and Chemistry, Faculdade de Ciências Farmacêuticas de Ribeirão Preto, São Paulo University-USP, Av. do Café s/n, Ribeirão Prêto, SP CEP-14.040-903 Brazil; 4 Faculdade de Ciências Farmacêuticas, Alimentos e Nutrição, Universidade Federal do Mato Grosso do Sul - UFMS, Av. Costa e Silva, s.n., Campo Grande, MS 79070900 Brazil; 5 Centro universitário SENAI-CIMATEC, Av. Orlando Gomes, 1845, Piatã, Salvador, BA 41650-010 Brazil

**Keywords:** Biochemistry, Biotechnology, Chemistry, Energy science and technology, Materials science, Nanoscience and technology

## Abstract

SiO_2_-SO_3_H, with a surface area of 115 m^2^/g, pore volumes of 0.38 cm^3^g^−1^ and 1.32 mmol H^+^/g, was used as a 10% w/w catalyst for the preparation of 5-hydroxymethyl-2-furfural (HMF) from fructose. A conversion of 100% was achieved in a microwave reactor during 10 min at 150 °C in DMSO, with 100% selectivity for HMF, at a molar ratio of fructose: DMSO equal to 1:56. The catalyst could be re-used three times.

## Introduction

**Figure 1:**
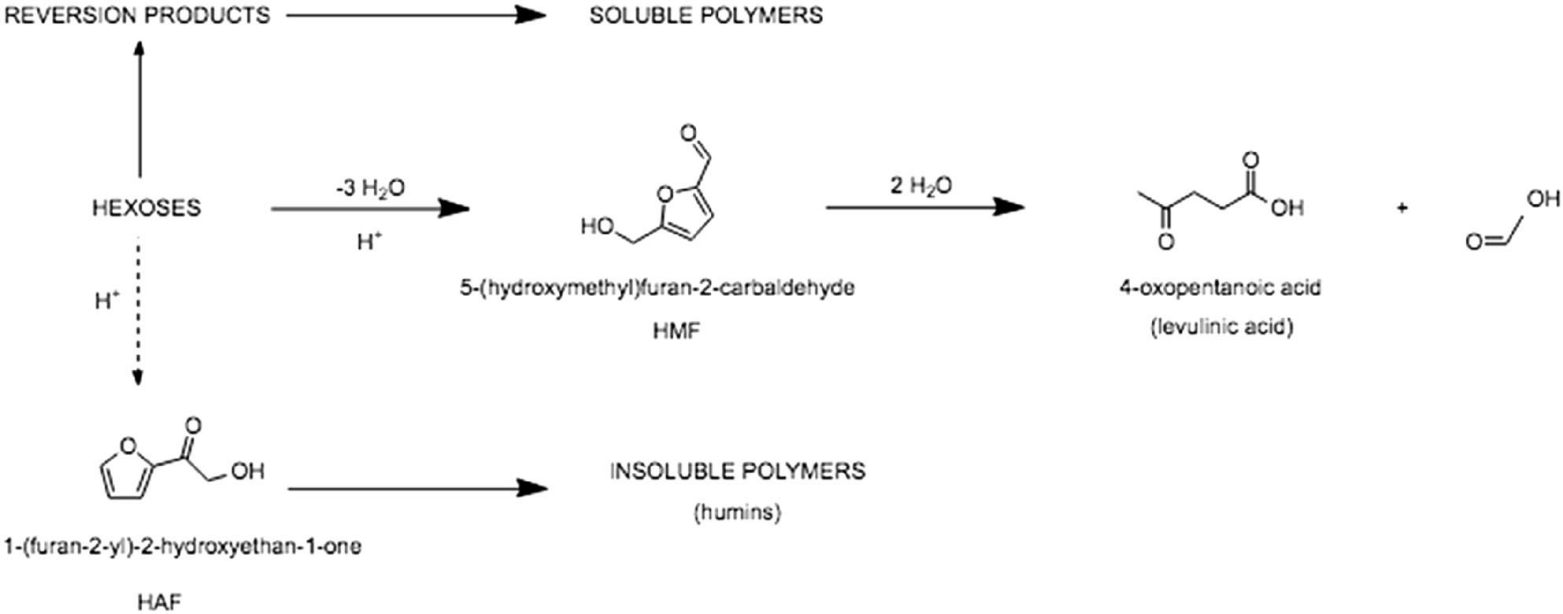
General pathways for the degradation of hexoses in water, catalysed by acids; variables such as concentration, temperature and use of co-solvent need to be strictly controlled to increase HMF selectivity and high hexoses conversion rates.

5-Hydroxymethyl-2-furfural (HMF), which can be obtained from the dehydration of sugars, has a high potential as renewable raw material for the production of a variety of important molecules containing or derived of the furan ring, including biofuels, solvents, drugs, and biopolymer monomers^1–3^. Thus, several studies have described the synthesis of HMF by catalytic dehydration of fructose^4–18^, Fig. 1. Glucose is less efficient than fructose for the synthesis of HMF^19–21^; according to Kuster^22,23^ and Zakrzewska et al.^24^, this results from the difference in stability of the two cyclical sugar structures, which are composed of six atoms in glucose (pyranose form) and five atoms in fructose (furanose form). The five-membered ring apparently facilitates the enolizations responsible for the generation of HMF. The main catalysts used for these conversions are mineral acids in aqueous solutions, such as H_2_SO_4_, HCl, or H_3_PO_4_^2,23^. However, the utilization of these systems suffers from several drawbacks; apart from the generally non-selectivity of the processes due to degradation of HMF via rehydration and polymerization reactions, the use of mineral acids involves material corrosion, difficulties in the separation of the acid from the reaction mixture, and, of course, the high toxicity of the acids themselves^8,25–28^. Therefore, the development of more environmentally friendly and convenient solid acid catalysts to replace the liquid acid catalysts is highly desirable.

The use of inorganic acids^25^, ionic liquids^24^, Lewis acids, metallic chlorides, oxides, phosphates, heteropoly acid compounds, mesoporous solids^3^ and ion exchange resins^29,30^ have been used as green solid acid catalysts for the formation of HMF. Although H‐form zeolites could be employed as solid acid catalysts for the dehydration of fructose and the reaction can be highly selective (60–90%), their practical use in the conversion of fructose into HMF has been largely limited by the lower conversion efficiency^31^. Materials such as reticulated polystyrene containing sulfonic groups^32,33^ and another polymeric heterogeneous catalyst such as polyaniline^24,34^ have also been studied.

Apart from the catalyst, the choice of solvent for the dehydration reaction is also very important. The commonly applied solvents are water, methanol, DMSO and ionic liquids^3,8,27,35^; water/organic solvent biphasic systems are also important to increase the conversion rates and selectivity, but the current methods require large amounts of solvents because of the high solubility of HMF in water and the poor partitioning into the organic phase, which sometimes requires additional salting-out techniques for the separation of the product^25^. In particular, anhydrous DMSO has such a strong tendency to associate with water molecules that it has been used to force transformations such as 2 R-OH + DMSO → R-O-R + DMSO·H_2_O, and accordingly, d-fructose was converted to HMF in pure DMSO kept at 150 °C for 2 h^36^.

As a part of an ongoing research on the use of the SiO_2_–SO_3_H catalyst for clean synthesis^37^, we report herein the preparation of HMF from fructose with DMSO as the solvent and the hydrophilic SiO_2_–SO_3_H catalyst using microwave irradiation in processes under ambient atmosphere; MW irradiation has been widely used in the production of HMF^17^, and recently, in its oxidation to value added acid derivatives using solid catalysts containing Ru^38^ or Ag^39^.

## Experimental

### Raw materials and chemicals

All reagents (analytical grade), including dry DMSO and d-Fructose were supplied by Vetec, São Paulo, Brazil.

### Instrumentation

HMF content and yield were determined with a GC/MS-QP 2010/AOC 5000 AUTO INJECTOR/Shimadzu Gas Chromatograph/Mass Spectrometer equipped with a 30 m Agilent J&W GC DB-5 MS column. Direct insertion spectra were measured at 70 eV. Quantitative analyses were performed on a Shimadzu GC-2010 gas chromatograph equipped with a flame ionization detector^37^. ^1^H- and ^13^C-NMR spectra were recorded on Bruker *Avance* 400 Spectrometers^37^. All reactions were monitored by TLC using Silica Gel 60 F 254 on aluminum. The chromatograms were visualized by UV light or by using the ethanolic vanillin developing agent^37^. The purification of the products was made by chromatographic column flash chromatography using a mixture of hexane/ethyl acetate in a 9/1 proportion as eluent^37^. The MW reactions were carried out in 10 mL G-10 vials of an Anton Paar single-mode MW synthesis reactor Monowave 300, powered by an 850 W magnetron, and equipped with temperature sensor and magnetic stirring.

### Preparation of the silica gel and sulfonated silica (SiO_2_–SO_3_H)

The preparation of silica gel and the sulfonated silica SiO_2_–SO_3_H catalyst have been reported previously^37^.

### Typical procedures

#### Dehydration of fructose using SiO_2_–SO_3_H as catalyst in DMSO

In a 10 mL microwave reactor vial, it was added 0.2604 mg (1.44 mmol) of D-fructose, 8.0 mL (8.80 g; 112.63 mmol) of DMSO and 0.0260 g (10% w/w in relation d-fructose) of SiO_2_–SO_3_H. The vial was heated in the microwave reactor at 150 °C for 10 min. The resulting dark liquid obtained after filtration of the solid catalyst was transferred to an extraction funnel and diluted with 30 mL of ethyl acetate and 30 mL of water. The lower fraction containing DMSO was removed and stored for future DMSO extraction and water purification, and the remaining organic solution was partitioned between 30 mL of ethyl acetate and 30 mL saturated NaCl, dried with magnesium sulfate, filtered and evaporated under reduced pressure. The resulting residue was subjected to a GC/MS analysis, which demonstrated the absence of unreacted d-fructose. The residue was then purified by flash column chromatography on silica, using hexane: ethyl acetate (2:1) as the mobile phase to yield HMF as a reddish-brown oil. Alternatively, the mixture of d-fructose, catalyst and DMSO were placed in an 100 mL round bottomed flask equipped with a condenser protected by a CaCl_2_ tube, and heated in a sand bath for 2 h; the treatment of the resulting dark liquid was identical to the described above.

## Results and discussion

**Scheme 1:**
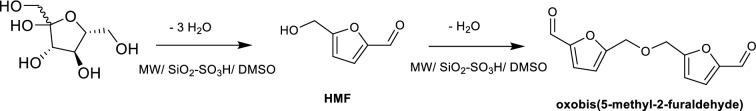
Reaction involved in the conversion of d-fructose to HMF using SiO_2_–SO_3_H.

In 1983 Musau and Munavu described the synthesis of HMF in 92% yield when d-fructose was dehydrated using DMSO as both a dehydrating agent and as the solvent at 150 °C for 2 h. In their work the optimum conversion occurred at a d-Fructose: DMSO molar ratio of 1:8.5. Under those reaction conditions the condensation of HMF itself becomes important, and 1.2% of oxobis (5-methyl-2-furaldehyde) was also obtained^36^. Seeking to improve the efficiency of this reaction and reduce the reaction time, we inserted the SiO_2_–SO_3_H catalyst^37^ into the reaction mixture, using both conventional and microwave heating. Scheme 1 summarizes the conditions employed in the dehydration of d-fructose.

Microwave irradiation was used as an energy source for reaction activation because of its advantages over conventional heating methods^14,15^. As expected, conventional heating of the reaction mixture required longer reaction times and furnished lower yields than microwave heating, except for Entry 7 in Table 1, where no solvent was added to the mixture of catalyst and HMF; Table 1 resumes the principal results of the present work.

**Table 1:** Conditions used to obtain HMF by dehydration of D-fructose at 150 °C.

	Catalyst SiO_2_–SO_3_H 10% w/w	Solvent DMSO	Type of heating and reaction time at 150 °C	Fructose conversion % by GC/MS	HMF Isolated yield %
1	No	Yes	Conventional, 2 h	(a)	92(a)
2	No	Yes	MW, 30 min	100	89
3	Yes	Yes	Conventional, 2 h	97(b)	90
4	Yes	Yes	MW, 10 min	100	91
5	Yes	Yes	MW, 15 min	100(c)	85
6	Yes	Yes	MW, 30 min	100(d)	84
7	Yes	No	MW, 30 min	–	–

(a) According to reference 36, plus 1.2% oxobis(5-methyl-2-furaldehyde); (b) 3% recovered fructose; (c) 98% plus 2% oxobis(5-methyl-2-furaldehyde); (d) 95% plus 5% oxobis(5-methyl-2-furaldehyde).

In Table 1, the first entry refers to the original work of Musau and Munavu, which used DMSO as the sole dehydrating agent, using conventional heating^36^. This work shows that, as expected, the use of MW irradiation speeds the overall process, and the excellent mark of 100% HMF conversion could be achieved in only 30 min, Entry 2.

The comparison between MW and conventional heating becomes more interesting when one considers the reactions involving the synergic dehydrating effects of the solvent DMSO and of the hydrophylic catalyst SiO_2_–SO_3_H, which had already been successfully used to dehydrate benzylic alcohol to dibenzyl ether^37^. Apparently, under conventional heating (Entry 3, Table 1) the combined effects are small, and the % results of the dehydration of fructose in the presence of the catalyst are comparable to Musau’s report. However, it is interesting to note that with the use of DMSO alone, Musau and Monavu observed the formation of 1.2% of the HMF condensation product (Entry 1), which was not observed in the same reaction performed in the presence of the sulfurous catalyst (Entry 3). It is possible that the catalyst increases the specificity of the DMSO towards the dehydration of the hexose.

Entry 4 in Table 1 suggests that the synergic DMSO/ SiO_2_–SO_3_H hexose dehydrating effect may be at its optimum level under MW irradiation, by showing that the excellent mark of 100% fructose conversion can be achieved again, but within only 10 min irradiation, with no products other than HMF being formed (by CG-MS analysis). This result compares very favorably with that of Watanabe et al.^40^, who found a very good 94% fructose conversion but a smaller 73% HMF yield, obtained by the catalytic dehydration of fructose in acetone–water mixtures in the presence of a sulfonic acid resin catalyst, at the same temperature of 150 °C, and under MW irradiation.

The specificity and dehydrating capacity of the DMSO/ SiO_2_-SO_3_H system was further tested increasing the contact times of the fructose conversion reactions, and Entries 5 and 6 in Table 1 show that condensation to oxobis(5-methyl-2-furaldehyde) indeed occurs after the hexose had been 100% converted to HMF. No other side-product was observed. The augment of the yield of the bis-furaldehyde ether from 1.2% (pure DMSO, conventional heating, ref.^36^) to 5% (DMSO/SiO_2_–SO_3_H/MW irradiation, this work) may indicate a possible, clean route for that interesting ether, but this route was not investigated further.

## Conclusions

The use of microwave irradiation decreased fourfold the time required for the production of HMF by dehydration of fructose with DMSO at 150 °C and allowed 100% conversion and 100% selectivity. Addition of the hydrophylic catalyst SiO_2_–SO_3_H to that system reduced the reaction time a further threefold, maintaining the excellent mark of 100% fructose conversion and 100% selectivity to HMF. The catalytic system seems to be promising for the preparation of oxobis (5-methyl-2-furaldehyde) ether from HMF.

## Supplementary Information

Supplementary Information.
