# Supplementary material for: Dehydration of d-fructose to 5-hydroxymethyl-2-furfural in DMSO using a hydrophilic sulfonated silica catalyst in a process promoted by microwave irradiation
Source: Sci Rep. 2021 Jan 21;11:1919. doi: 10.1038/s41598-020-80285-2 (PMC7820282; doi:10.1038/s41598-020-80285-2)
Supplement: Supplementary file 1 — Supplementary Information. [file 41598_2020_80285_MOESM1_ESM.docx]

**Dehydration of D-fructose to 5-hydroxymethyl-2-furfural in DMSO using a hydrophilic sulfonated silica catalyst in a process promoted by microwave irradiation**

Sandro L. Barbosa,^a*^ Milton de S. Freitas,^a^ Wallans T. P. dos Santos,^a^ David Lee Nelson,^a^ Stanlei I. Klein,^b^ Giuliano Cesar Clososki,^c^ Franco J. Caires,^c^ Adriano C. M. Baroni^d^, Alexandre P. Wentz^e^

^a^Department of Pharmacy, Universidade Federal dos Vales do Jequitinhonha e Mucuri-UFVJM, Campus JK, Rodovia MGT 367 - Km 583, nº 5.000, Alto da Jacuba, CEP 39100-000, Diamantina/MG, Brazil. e-mail: [sandro.barbosa@ufvjm.edu](mailto:sandro.barbosa@ufvjm.edu); [freitas.milton@hotmail.com](mailto:freitas.milton@hotmail.com); wallanst@yahoo.com.br; dleenelson@gmail.com

^b^Department of General and Inorganic Chemistry, Institute of Chemistry, São Paulo State University-Unesp, R. Prof. Francisco Degni 55, Quitandinha, CEP-14.800-900 Araraquara/SP, Brazil; e-mail: [stanleiklein@gmail.com](mailto:stanleiklein@gmail.com)

^c^Department of Biomolecular Sciences, Faculdade de Ciências Farmacêuticas de Ribeirão Preto, São Paulo University-USP, Av. do Café s/n, CEP-14.040-903 Ribeirão Preto/SP, Brazil; e-mail: gclososki@usp.br; [fjcaires@usp.br](mailto:fjcaires@usp.br)

^d^Faculdade de Ciências Farmacêuticas, Alimentos e Nutrição, Universidade Federal do Mato Grosso do Sul - UFMS, Av. Costa e Silva, s.n., Campo Grande, MS, 79070900, Brazil, e-mail: adriano.baroni@ufms.br

^e^Centro universitário SENAI-CIMATEC, Av. Orlando Gomes, 1845, Piatã, 41650-010, Salvador, BA, Brazil; e-mail: alexandre.wentz@fieb.org.br

*Corresponding author. Tel.: +55-38-35321234; fax: +55-38-35321234; e-mail: [sandro.barbosa@ufvjm.edu.br](mailto:sandro.barbosa@ufvjm.edu.br)

_____________________________________________________________________________________

**Abstract**: SiO_2_-SO_3_H, with a surface area of 115 m^2^/g, pore volumes of 0.38 cm^3^g^-1^ and 1.32 mmol H^+^/g,

was used as a 10% w/w catalyst for the preparation of 5-hydroxymethyl-2-furfural (HMF) from fructose. A conversion of 100% was achieved in a microwave reactor during 10 minutes at 150 ^o^C in DMSO, with 100% selectivity for HMF, at a molar ratio of fructose: DMSO equal to 1:56. The catalyst could be re-used three times.

*Keywords:* Sulfonated silica catalyst; DMSO, 5-hydroxymethyl-2-furfural, fructose, dehydration.

_____________________________________________________________________________________

**Characterization data**

HMF: liquid at r.t. GC/MS - m/z (%): 97 (100) [M-CHO]^+^ C_5_H_5_O_2_^+^, 109 (20) [M-OH]^+^ C_6_H_5_O_2_^+^, 126 (80) [M]^+^ C_6_H_6_O_3_. ^1^H-NMR [400 MHz, CDCl_3_, δ (ppm)] of HMF: 4.52 (CH_2_OH), 6.51 (-OH) , 6.60 (C^4^H, d, J = 3.5 Hz), 7.10 (ring C^3^H, d, J = 3.5 Hz) , 9.51 (CHO, 1H, s). ^13^C NMR (CDCl_3_, δ (ppm), 100-MHz): 177.6, 161.6, 152.2, 121.9, 109.7, 57.3.

Copies of ^1^H and ^13^C NMR spectra for HMF compounds, plus CG/MS.

1) HMF


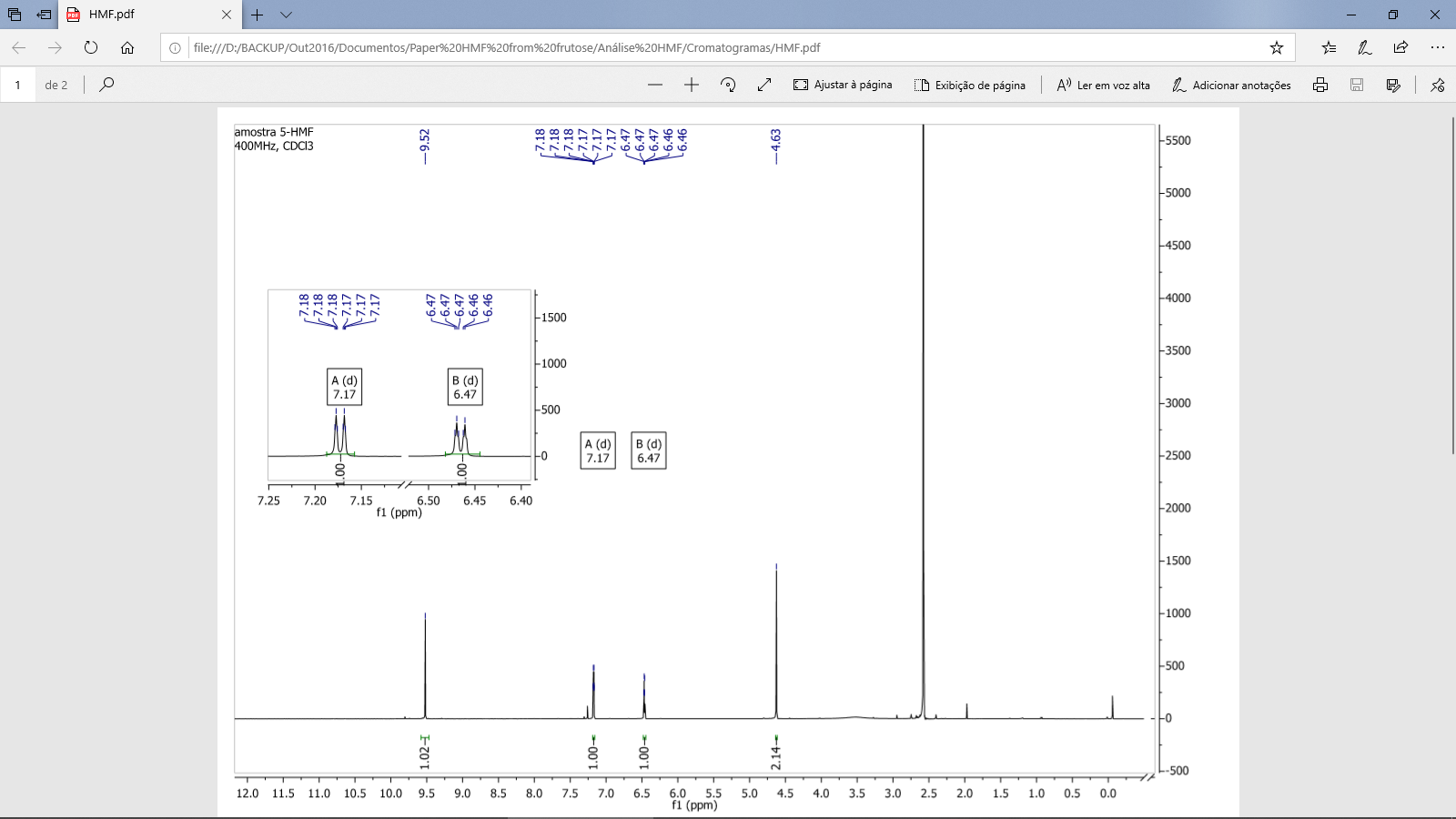


Figure S1. ^1^H-NMR [400 MHz, CDCl_3_, δ (ppm)] of HMF: 4.52 (CH_2_OH), 6.51 (-OH) , 6.60 (C^4^H, d, J = 3.5 Hz), 7.10 (ring C^3^H, d, J = 3.5 Hz) , 9.51 (CHO, 1H, s).


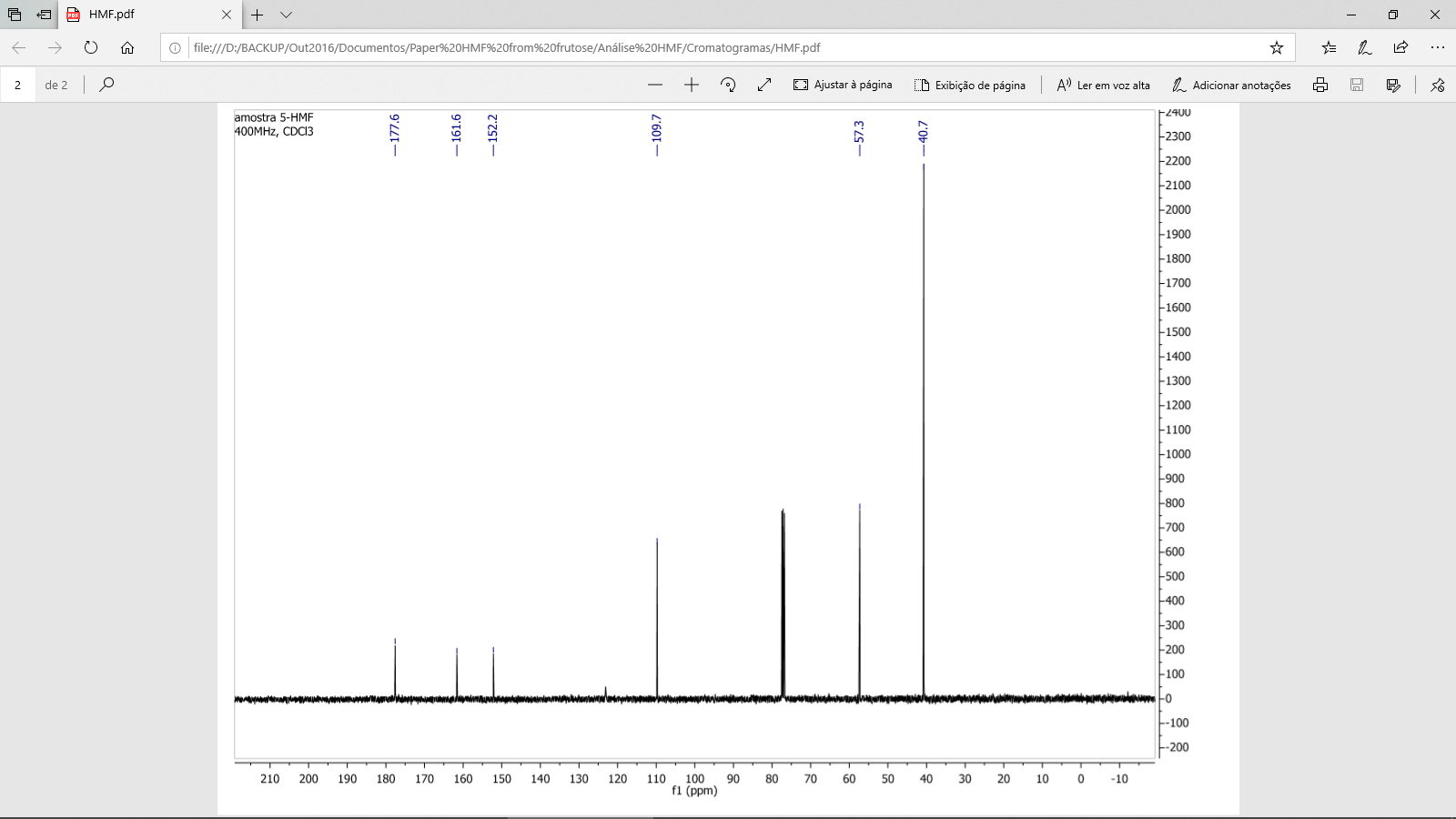


Figure S2. ^13^C NMR [100 MHz, CDCl_3_, δ (ppm)] of HMF: 177.6, 161.6, 152.2, 121.9, 109.7, 57.3.


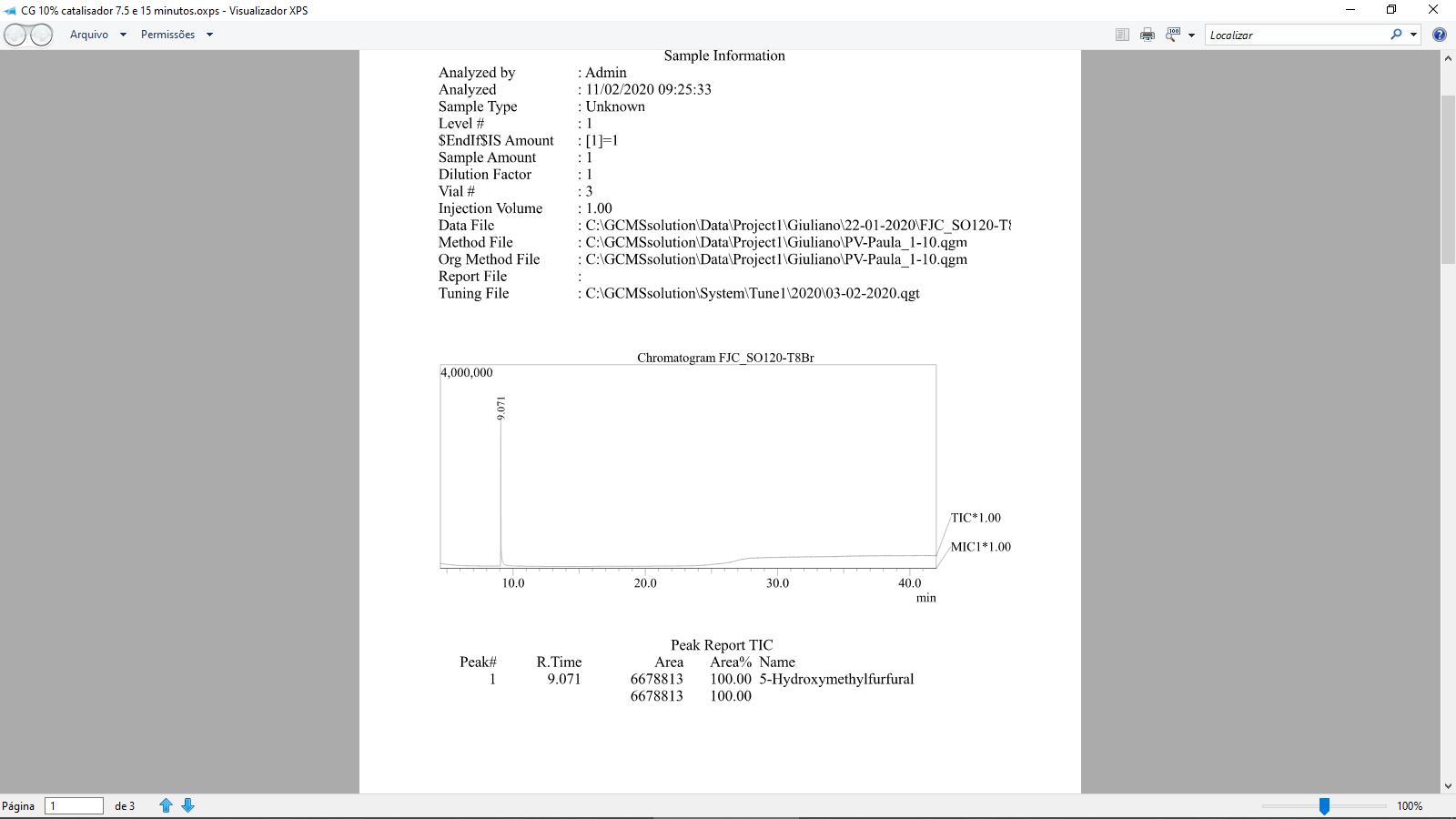


Figure S3. GC Chromatogram of HMF.


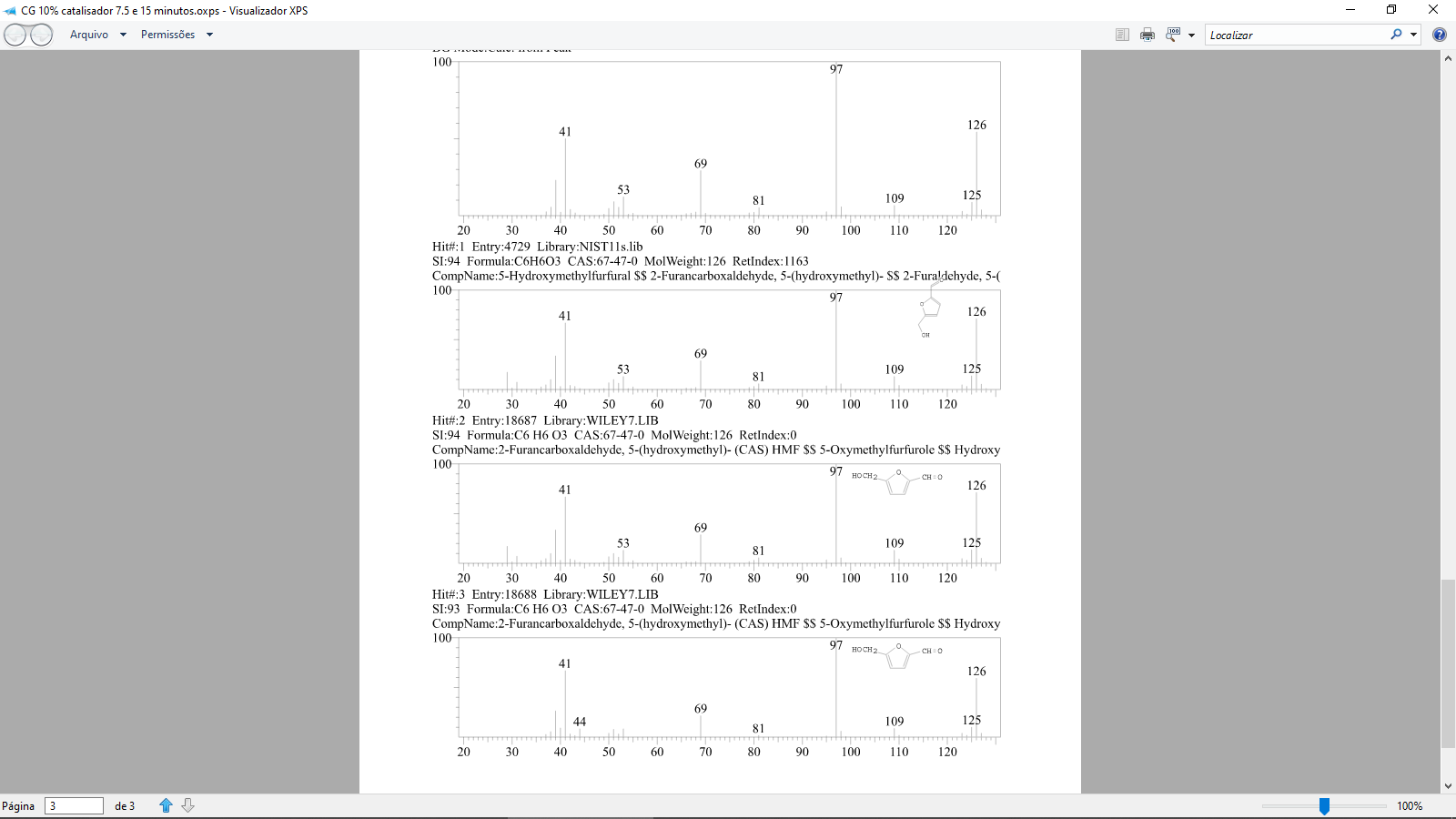


Figure S4. Mass spectrum of HMF, m/z (%): 97 (100) [M-CHO]^+^ C_5_H_5_O_2_^+^, 109 (20) [M-OH]^+^ C_6_H_5_O_2_^+^, 126 (80) [M]^+^ C_6_H_6_O_3_.
